# Induction of Different Apoptosis Pathways by Two *Proteus* *mirabilis* Clinical Isolates Strains in Prostatic Epithelial Cells

**DOI:** 10.3389/fphys.2018.01855

**Published:** 2018-12-20

**Authors:** Alessandra Fusco, Vittoria Savio, Anna De Filippis, Antonio Tufano, Giovanna Donnarumma

**Affiliations:** ^1^Department of Experimental Medicine, Section of Microbiology and Clinical Microbiology, University of Campania Luigi Vanvitelli, Naples, Italy; ^2^Urology School, Sapienza University of Rome, Rome, Italy

**Keywords:** *Proteus*, UTIs, prostatitis, apoptosis, antibiotic resistance

## Abstract

Bacterial prostatitis is believed to be the leading cause of recurrent urinary tract infections (UTIs) in men under 50 years of age and occurs both as an acute febrile disease responsive to antibiotics and as a chronic infection that is often unresponsive to antibiotic treatment. *Proteus* *mirabilis* is more commonly associated with UTIs in these abnormalities, especially in patients undergoing catheterisation. This pathogen is able to colonise the host’s tissues and to cause disease thanks to the production of many virulence factors such as fimbriae, flagella, immune avoidance, host-damaging factors, and the ability to form crystalline biofilms. In addition, *Proteus* lipid A may exhibit apoptotic activity and induce desquamation of epithelial cells. The aim of this work was to evaluate the ability of two clinically isolated strains of *P. mirabilis* that are phenotypically different, named PM1 of PM2, respectively, to induce apoptosis in human prostatic adenocarcinoma PC-3. Our results demonstrate that PM1 and PM2 are able to activate two different apoptotic pathways, and this different behaviour is confirmed by the expression level of the *ZapA* gene, molecular fingerprinting and different spectrum of antibiotic resistance. The identification and knowledge of relations between the microorganism and host may provide the basis for new solutions to clinical problems with regard to diagnosis and therapy.

## Introduction

Bacterial prostatitis is a pathological condition that can manifest with varying clinical features, including inflammation, pelvic pain (localised to the prostate, perineum, or urethra), problems with voiding, and a variable degree of sexual dysfunction. In particular, this disease also involves psychological aspects along with the onset of depressive syndromes due to the persistence of intense pain and the frequency of urination, resulting in the worsening of a patient’s quality of life (Chen et al., 2014; Krsmanovic et al., 2014; Sandhu and Tu, 2017).

Prostate involvement is observed in more than 90% of febrile urinary tract infections (UTIs) (Edefonti et al., 2014; Schaeffer and Nicolle, 2016), and can manifest in the following two forms: an acute form that responds to antibiotic treatment and whose symptoms persist for a time interval between 1 and 3 months and a chronic form that is often associated with biofilm formation with recurrent symptoms and which does not respond to antibiotic treatments. The resistance is caused by the inability of antibiotic to penetrate through the prostatic epithelium at concentrations sufficient to carry out their bactericidal activity.

An important risk factor for UTIs and bacterial prostatitis is represented by benign prostatic hyperplasia (BPH) (Bradway et al., 2013; Wagenlehner et al., 2014), a structural abnormality that affects, mainly, men of and above 60 years of age (Zhang et al., 2013). A prostate enlargement that prevents complete bleeding of the bladder, resulting in increased bacterial growth and the risk of developing an infection, is observed in BPH (Bradway et al., 2013).

*Proteus* *mirabilis* is one of the most causative agents commonly associated with UTIs, especially in patients undergoing urinary catheterisation. This gram-negative bacillus is known for its ability to show a typical movement called swarming migration on solid media, which occurs after the cell differentiation process that involves the transformation of typical vegetative rods (2 to 4 μm long) into long filaments that are multinucleated with numerous flagella arranged along its entire surface (Schaffer and Pearson, 2015).

*Proteus mirabilis* is capable of colonising a host and causing disease due to its arsenal of virulence factors, including the formation of crystalline biofilm, fimbriae, capsule, lipopolysaccharide (LPS), and various enzymes. Amongst these, that role of ZapA as a virulence factor has been recently demonstrated in a rat model of *P. mirabilis*-induced prostatitis and progression to chronic infection (Belas et al., 2004; Carson et al., 2012). This enzyme belongs to the serralysin extracellular metalloproteases, which is also called IgA protease (Belas et al., 2004; Carson et al., 2012), which uses zinc and divalent cations as cofactors (Fukasawa et al., 2011) and may degrade a number of substrates, including components of the host’s immune system, such as immunoglobulin A (IgA), complement factors, antimicrobial peptides, and cytokines. The expression of ZapA is closely related to swarmer cell differentiation and swarming behaviour (Cusick et al., 2012). Indeed, it is ca. 30 times greater in swarmer cells than in uninduced swimmer cells. Moreover, it is known that swarmer cell formation in *P. mirabilis* is associated with biofilm formation (Fusco et al., 2017), which is a complication in the pathogenesis of UTIs. *P. mirabilis* can produce biofilms both on non-living surfaces (such as glass polystyrene and silicone) and on biological surfaces. This crystalline biofilm is composed of two main types of crystals, named struvite (crystals of magnesium ammonium phosphate) and apatite (microcrystalline structures of hydroxylated calcium phosphate or carbonate), and allows the microorganism to reproduce and grow in the urinary tract, as it protects it from antibiotics and from the host’s immune defences. The persistence of biofilm can lead to the development of bladder stones and to accumulation of ammonia which can induces tissue damage because of its toxicity towards the uro-epithelial cell. In addition, *Proteus* lipid A induces desquamation of epithelial cells and may have apoptotic activity cells (Foris and Snowden, 2017). The metalloproteases ZapA of *P. mirabilis* and LasB of *Pseudomonas aeruginosa* are considered virulence factors for these opportunistic bacterial pathogens as during the infectious process, they contribute to create tissue damage by inactivating the components of the host’s immune system. Therefore, their inhibition may represent an antimicrobial strategy, to mitigate the virulence of the infecting pathogen (Carson et al., 2012).

In our previous study (Fusco et al., 2017), we characterised two clinically isolated strains of *P. mirabilis*, named PM1 and PM2, respectively, demonstrating that they have a different profile that is both biochemical, such as the different speeds in biofilm formation and the ability to differentiate in swarmer cells, and molecular, as shown after the amplification of 16S-23S RNA and the after performing an analysis of the expression levels of *WosA* and *flhDC* genes, which are closely related with the formation of biofilm. We also showed that the two strains were able to induce a different inflammatory response in bladder epithelial cells.

Our results highlighted the necessity of accurately diagnosing urinary infections to individuate the different bacterial strains and to choose an appropriate antibiotic therapy. Therefore, in this study, we have deepened the differences between *P. mirabilis* PM1 and PM2 through genetic fingerprinting and the analysis of the expression of virulence factor *ZapA* and also by evaluating the response induced in prostate epithelial cells in terms of cytotoxicity and the induction of apoptosis.

## Materials and Methods

### Bacterial Strains and Antimicrobial Susceptibility Assay

The *P. mirabilis* strains (PM1 and PM2) were isolated, as previously reported in our work (Fusco et al., 2017), and were grown in the Luria-Bertani (LB) medium at 37°C in aerobic conditions.

*In vitro* susceptibility testing was performed on all isolates and interpreted using Phoenix^TM^ (BD Diagnostic Systems) for the following antibiotics: ceftazidime, amikacin, tobramycin, ciprofloxacin, gentamicin, piperacillin, tazobactam-piperacillin, colistin, cefepime, levofloxacin, meropenem, ampicillin, cefotaxime, ertapenem, cefuroxime, fosfomycin c/G6P, trimethoprim-sulfamethoxazole, amoxicillin-clavulanic acid, and tigecycline. The minimum inhibitory concentration values that were obtained using the above methods were categorised according to the National Committee for Clinical Laboratory Standards’ breakpoints as susceptible (S), intermediate (I), or resistant (R).

### DNA Fingerprinting and Evaluation of *ZapA* Expression

For DNA isolation, single colonies of both PM1 and PM2 were inoculated on the LB medium and grown overnight at 37°C. Subsequently, 2 ml of each sample were used for DNA extraction using the QIAamp DNA Mini Kit (Qiagen) in accordance with the manufacturer’s protocol.

Random amplified polymorphic DNA (RAPD)-PCR analysis and *ZapA* evaluation were performed in a GeneAmp PCR System 9700 (Applied Biosystems) and hot start GoTaq Polymerase (Promega). The amplification programs of RAPD included the following: initial denaturation at 92°C for 4 min, followed by 40 cycles of 1 min at 94°C, 1 min at 40°C, 5 min at 72°C, and a final extension for 5 min at 72°C. The sequences of the primers used are shown in Table 1.

**Table 1:** Primers sequence for RAPD-PCR.

Gene	Sequence	Total number of bands	Total number of the band in Proteus	Number of the polymorphic band in Proteus	Simpson’s diversity index (DI)
OPA10	5′-GTGATCGCAG-3′	23	5	3	0.756
OPA11	5′-CAATCGCCGT-3′	31	12	9	0.899
OPD20	5′-ACCCGGTCAC-3′	9	3	2	0.333
OPX13	5′-ACGGGAGCAA-3′	23	13	9	0.921
OPX15	5′-CAGACAAGCC-3′	15	8	2	0.563
OPZ04	5′-AGGCTGTGCT-3′	11	7	1	0.335
OPZ08	5′-GGGTGGGTAA-3′	15	10	8	0.884
OPZ10	5′-CCGACAAACC-3′	16	7	5	0.627
OPZ19	5′-GTGCGAGCAA-3′	26	14	7	0.847
OPZ20	5′-ACTTTGGCGG-3′	19	7	5	0.945
		188	86	51	0.998


For *ZapA* PCR, the primers were as following: *ZapA* for 5′-ACCGCAGGAAAACATATAGCCC-3 and *ZapA* rev 5′-GCGAC TATCTTCCGCATAATCA-3 (533 bp). The amplification program involved initial denaturation at 95°C for 5 min, followed by 35 cycles of 1 min at 95°C for 1 min at 53°C, 1 min at 72°C, and a final extension of 5 min at 72°C.

The amplification products were analysed on 1.6% agarose gels to control the amplicon lengths in Tris-borate-EDTA buffer (0.089M Tris, 0.089M boric acid, 0.002M EDTA).

### Skimmed Milk Assay

PM1 and PM2 were plated on Skim Milk Agar consisting in skim milk powder 28 g, tryptone 5 g, yeast extract 2.5 g. dextrose 1 g and agar 15g per litre of dH_2_O and incubated overnight at 37°C (Wassif et al., 1995; Walker et al., 1999). The positivity of the reaction is revealed with the appearance of a transparent zone around the colonies, a sign of the presence of a protease activity.

### Cell Culture and Treatment

PNT-2 (normal human prostate epithelial cells- Sigma-Aldrich; data not shown) and PC3 cells (human prostatic adenocarcinoma- ATCC) (Yang et al., 2013; Gao et al., 2014; Shan et al., 2014) were cultured in RPMI (Gibco) that was supplemented with 1% Penstrep, 1% glutamine, and 10% foetal calf serum (Invitrogen) at 37°C in air and 5% CO_2_. Subsequently, the cells were dispensed into 6-well plates and left to grow until 80% of confluence (Fusco et al., 2017).

Semiconfluent monolayers were then infected with exponentially growing PM1 and PM2 at a multiplicity of infection (MOI) of five bacteria/cell. The process of infection was carried out for 18 (for gene expression) and 24 (for ELISA assay) hours at 37°C in 5% CO_2_.

In order to evaluate the expression of apoptotic genes, the cells were then washed three times with sterile PBS, and total RNA was extracted using High Pure RNA Isolation Kit (Roche Diagnostics).

### Real-Time PCR

A total of 200 nanograms of total cellular RNA were reverse-transcribed (Expand Reverse Transcriptase, Roche) into complementary DNA (cDNA) using random hexamer primers (Random Hexamers, Roche) at 42°C for 45 min, according to the manufacturer’s instructions (Fusco et al., 2017).

Real-time PCR for *Bcl-2*, *BAX*, *caspase-9*, *p53*, *TNFR1*, *TNF-α*, *Fas-L*, *Fas-R*, and *caspase-8*, -*3* and -*6* was carried out with the LC Fast Start DNA Master SYBR Green kit using 2 μl of cDNA that corresponds to 10 ng of total RNA in the final volume of 20 μl, 3 mM MgCl2, and 0.5 mM sense and antisense primers (Table 2).

**Table 2:** Primers sequence for real-time PCR.

	Primers sequence	Conditions	Product size (bp)
BAX	5′-TGGCAGCTGACATGTTTTCTGAC-3′ 5′-CGTCCCAACCACCCTGGTCT-3′	5″ at 94°C, 4″ at 56°C, 8″ at 72°C for 40 cycles	200
Bcl-2	5′-CAGCTGCACCTGACGCCCTT-3′ 5′-CCCAGCCTCCGTTATCCTGGA-3′	5″ at 94°C, 7″ at 58°C, 9″ at 72°C for 40 cycles	235
TNF-R1	5′-ACCAAGTGCCACAAAGGAAC-3′ 5′-CTGCAATTGAAGCACTGGAA-3′	5″ at 95°C, 5″ at 53°C, 10″ at 72°C for 40 cycles	263
Fas-L	5′-GGATTGGGCCTGGGGATGTTTCA-3′ 5′-TTGTGGCTCAGGGGCAGGTTGTTG-3′	5″ at 95°C, 7″ at 60°C, 14″ at 72°C for 40 cycles	344
Fas-R	5′-CCAAGTGACTGACATCAACTC-3′ 5′-CTCTTTGCACTTGGTGTTGCTGG-3′	5” at 94 °C, 8” at 55 °C, 17” at 72 °C for 40 cycles	426
p53	5′-TTCTTGCATTCTGGGACAGCC-3′ 5′-GCCTCATTCAGCTCTCGGAAC-3′	5” at 94 °C, 13” at 56 °C, 26” at 72 °C for 40 cycles	650
Caspase-3	5′-TTAATAAAGGTATCCATGGAGAACACT-3′ 5′-TTAGTGATAAAAATAGAGTTCTTTTGTGAG-3′	5” at 94 °C, 17” at 55 °C, 33” at 72 °C for 40 cycles	838
Caspase-6	5′-GGACCACAGGAGGAGAGGAATTGC-3′ 5′-GCACATCGTGCTGGTTTCCCCGAC-3′	5” at 94 °C, 6” at 59 °C, 13” at 72 °C for 40 cycles	317
Caspase-8	5′-CTGCTGGGGATGGCCACTGTG-3′ 5′-TCGCCTCGAGGACATCGCTCTC-3′	5” at 94 °C, 15” at 60 °C, 15” at 72 °C for 40 cycles	380
Caspase-9	5′-TTCCCAGGTTTTGTTTCCTG-3′ 5′-CCTTTCACCGAAACAGCATT-3′	5” at 94 °C, 3” at 55 °C, 6” at 72 °C for 40 cycles	143
TNF-α	5′-CAGAGGGAAGAGTTCCCCAG-3′ 5′-CCTTGGTCTGGTAGGAGACG-3′	5″ at 95°C, 6″ at 57°C, 13″ at 72°C for 40 cycles	324


At the end of each run, the melting curve profiles were achieved by cooling the sample to 65°C for 15 s and then heating it slowly at 0.20°C/s up to 95°C with continuous measurement of fluorescence to confirm the amplification of specific transcripts. Cycle-to-cycle fluorescence emission readings were monitored and analysed using LightCycler^®^ software (Roche Diagnostics GmbH). Melting curves were generated after each run to confirm the amplification of specific transcripts. We used the β-actin coding gene, one of the most commonly used housekeeping genes, as an internal control gene. All reactions were carried out in triplicate, and the relative expression of a specific mRNA was determined by calculating the fold change relative to the β-actin control. The fold change of the tested gene mRNA was obtained with a LightCycler^®^ software, by using the amplification efficiency of each primer, as calculated by the dilution curve.

The specificity of the amplification products was verified by subjecting the amplification products to electrophoresis on 1.5% agarose gel and visualisation by ethidium bromide staining (Fusco et al., 2017).

### ELISA Assay

PC3 cell monolayers were infected with PM1 and PM2 for 24 h at 37°C, as described above. At the end of the experiment, cellular lysates or supernatants were used to analyse the production of BAX, Bcl-2, caspase-6, Fas-L and TNF-α by enzyme-linked immunosorbent assay (ELISA; Abcam, United Kingdom).

### MTT Assay

PNT2 (data not shown) and PC3 cells were seeded at a density of 1 × 10^3^/well in 96-well culture dishes. After 24 h, the cells were treated with PM1 and PM2 at an MOI of 5 bacteria/cell for 18 h and then incubated with MTT (0.5 mg/ml; Sigma) at 37°C for four 4 h and, subsequently, with DMSO at room temperature for 5 min. The spectrophotometric absorbance of the samples was determined by using Ultra Multifunctional Microplate Reader (Bio-Rad) at 570–655 nm (Mosmann, 1983).

### Statistical Analysis

The significant differences among the groups were assessed through two-way ANOVA by using GraphPad Prism 6.0. The data are expressed as means ± standard deviation (SD) of three independent experiments.

## Results

### Molecular Fingerprinting by Random Amplified Polymorphic DNA (RAPD)-PCR and Antimicrobial Susceptibility Assay

**FIGURE 1:**
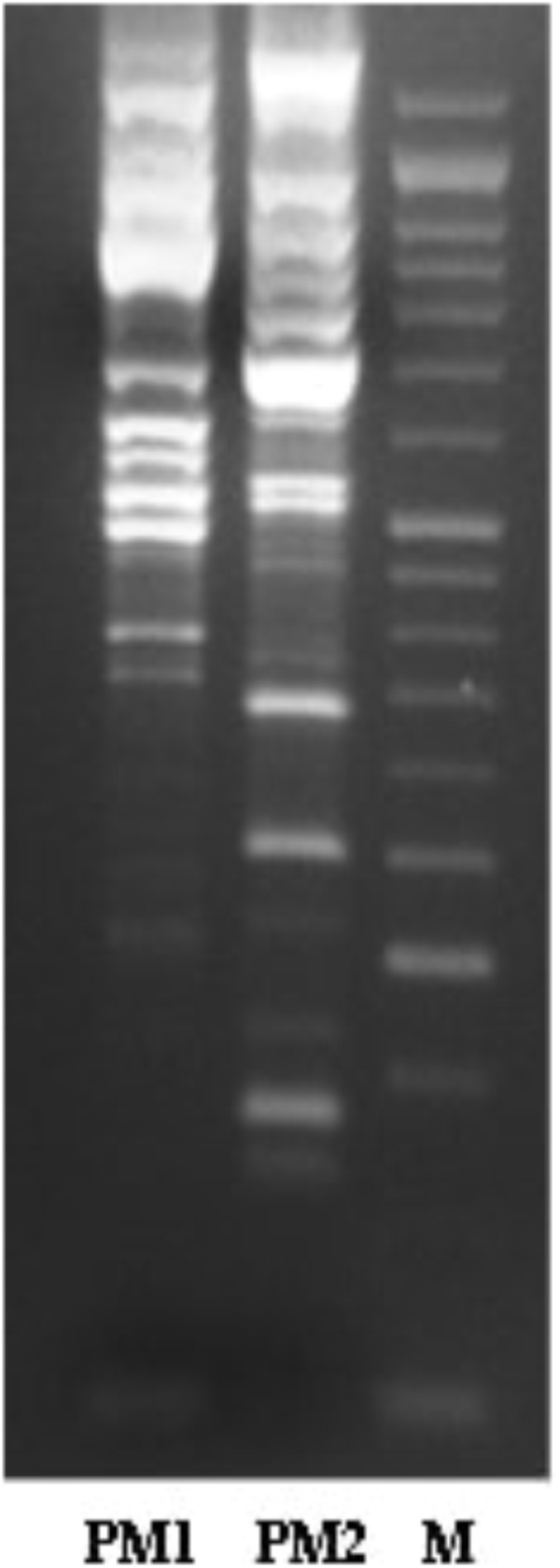
Molecular profiles obtained by RAPD-PCR for PM1 and PM2.

The characterisation of PM1 and PM2 by RAPD-PCR and by antimicrobial susceptibility assay, was performed in order to highlight the difference between the two strains both under the molecular profile and antibiotic resistance spectrum. In fact, RAPD-PCR revealed a banding pattern ranging from 0.2 to 3 kb, which displayed a high degree of variability (Figure 1). In addition, PM1 and PM2 have proven to possess a significant degree of diversity in terms of resistance to various classes of antibiotics, especially versus Amoxicillin-Clavulanic acid, Cefuroxime and Fosfomycin c/G6P, all of which are frequently used in the treatment of UTIs (Table 3). These data support the results based on the different behaviour of the two strains.

**Table 3:** Antimicrobial susceptibility profiles of PM1 and PM2 isolates when interpreted using Phoenix^TM^ (BD diagnostic systems).

	PM1		PM2	
ANTIBIOTIC	MIC/Conc.	SIR	MIC/Conc.	SIR
Amikacin	8	S	<=4	S
Amoxicillin-Clavulanic acid	<=2/2	S	>32/2	R
Ampicillin	<=2	S	>8	R
Cefepime	<=1	S	<=1	S
Cefotaxime	<=1	S	<=1	S
Ceftazidime	<=0.5	S	<=0.5	S
Cefuroxime	<02	S	>8	R
Ciprofloxacin	<=0.25	S	<=0.25	S
Colistin	>4	R	>4	R
Ertapenem	<=0.25	S	<=0.25	S
Fosfomycin c/G6P	<=16	S	64	R
Gentamicin	4	I	2	S
Levofloxacin	<=0.5	S	<=0.5	S
Meropenem	<=0.5	S	<=0.5	S
Piperacillin	<=4	S	<=4	S
Piperacillin-tazobactam	<=4/4	S	<=4.4	S
Tigecycline		R		R
Tobramycin	4	I	2	S
Trimethoprim-sulfamethoxazole	<=1/19	S	<=1/19	S


*The minimum inhibitory concentration values obtained were categorised according to the National Committee for Clinical Laboratory Standards’ breakpoints as susceptible (S), intermediate (I), or resistant (R).*

### Evaluation of *ZapA* Expression

**FIGURE 2:**
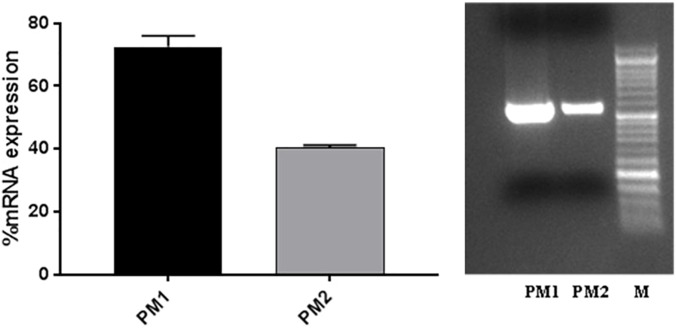
*ZapA* gene expression in PM1 and PM2 growth in LB broth; data are expressed as percentages of mRNA levels in each sample normalised to 16S RNA. Data are mean ± SD.

The evaluation of *ZapA* expression was performed, as this gene is considered to be one of the main virulence factors of *P. mirabilis*. The results obtained by RT-PCR show that *ZapA* is expressed as a percentage that is greater than 87.5% in PM1 compared with PM2 (Figure 2).

### Protease Activity

**FIGURE 3:**
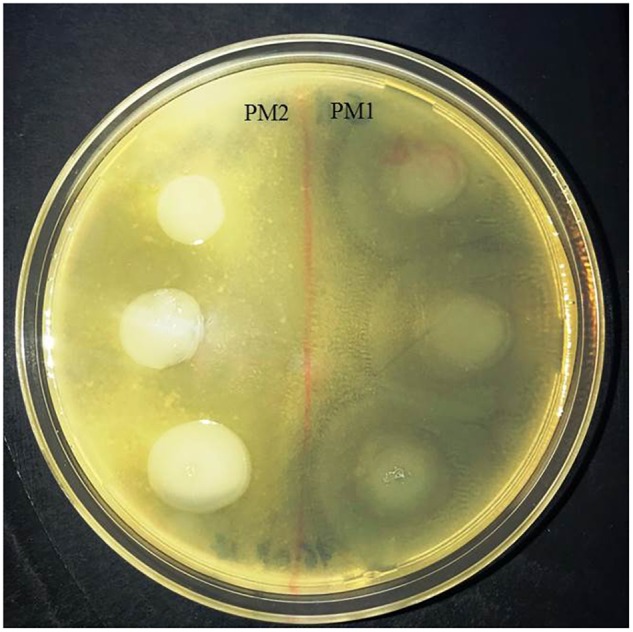
Skimmed milk agar test. Around the colonies of only PM1 large clear zone, due to the proteolysis of casein, are clearly visible.

In order to evaluate the protease activity of ZapA, the two strains PM1 and PM2 were plated on Skim milk agar. After incubation overnight at 37°C, PM1 colonies are surrounded by a wide transparent zone, while PM2 does not show any type of protease activity (Figure 3).

### Activation of Apoptotic Pathways and Cell Viability Measurement

**FIGURE 4:**
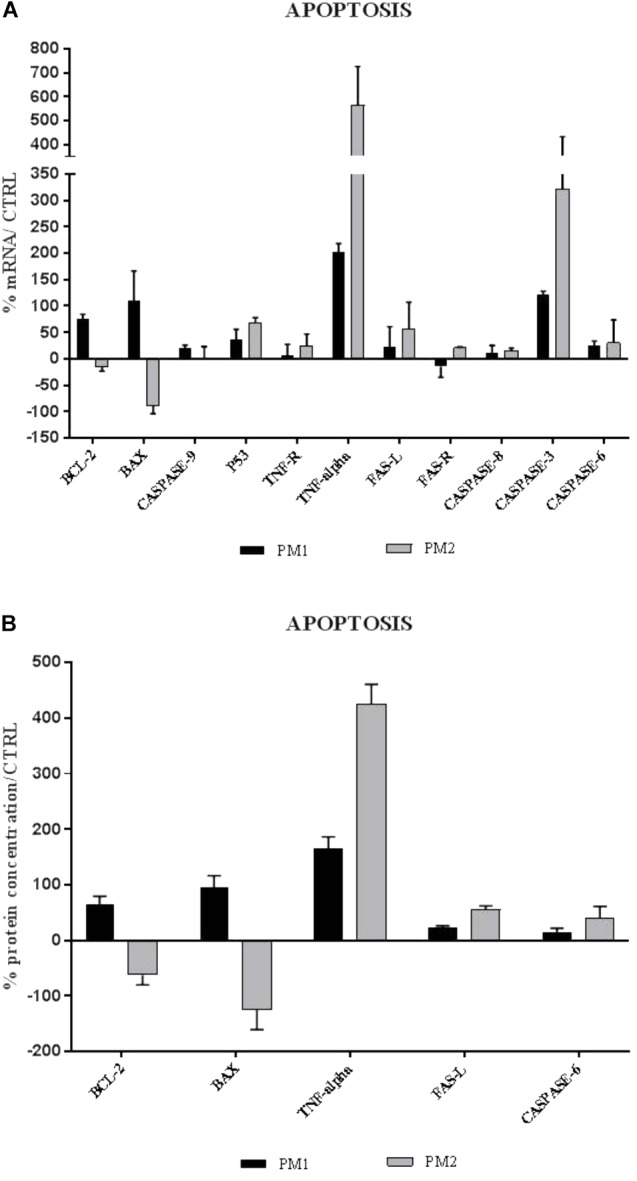
Comparison between relative gene expression **(A)** and protein concentration **(B)** of apoptotic factors in PC3 cells with PM1 and PM2. Data are mean ± SD and are expressed as percentages of mRNA levels in each group compared to unstimulated cells (CTRL).

The apoptosis gene expression analysis has shown that PM1 and PM2 activate two different apoptotic pathways. In fact, PM1 upregulates the intrinsic pathway factors (BCL-2, BAX, and Caspase-9) and, mainly, BCL-2, which is an anti-apoptotic factor that indicates the lowest degree of apoptosis induced by this strain. Conversely, PM2 modulates positively the expression of the genes that encode the apoptotic factors of the extrinsic pathway, whereas the genes that belong to the execution pathway are upregulated by both strains, even though modulation is much more apparent in PM2 (Figure 4A). Data were confirmed by ELISA assay (Figure 4B).

The assessment of cell viability was conducted to reinforce the apoptosis data. In fact, the results obtained from the MTT assay on uninfected, infected with PM1, and infected with PM2 cells show that after 18 h of incubation, the number of live cells remained substantially unchanged after PM1 infection with respect to control, whereas PM2 infection was capable of inducing a high level of cell death (Table 4).

**Table 4:** MTT assay: spectrophotometric absorbance values at 570 nm.

SAMPLE	O.D.
PC-3	2.9
PC-3 + PM1	2.613
PC-3 + PM2	0.733


## Discussion

One of the most common consequences of recurrent UTIs in men is represented by bacterial prostatitis (Wagenlehner et al., 2014). In this syndrome, whose incidence in the community is around 0.9 to two cases per 1000 men who are younger than 55 years and 7.7 cases per 1000 of men who are aged 85 years and above (Caljouw et al., 2011; Delcaru et al., 2017), bladder bacteriuria episodes can cause accumulation of bacteria in the prostate, resulting in the development of infection. This process is favoured by the appearance of prostate dysfunction, which begins to occur with the advancement of age, the most common symptom of which is BPH.

One of the most critical aspects in this type of infection is the importance of obtaining a correct diagnosis to set up an appropriate and effective therapy, so as to avoid the development of antibiotic resistance phenomena, which are now very common emerging problems (Gupta and Bhadelia, 2014).

In the first part of our work, we have deepened the characterisation, which had already begun in our previous study (Fusco et al., 2017), of two clinical isolation strains of *P. mirabilis*, a Gram-negative bacillus that is considered to be one of the major agents causing UTIs, shortly after *Escherichia coli*.

The two strains, named PM1 and PM2, respectively, first showed a very different antibiotic resistance spectrum. PM1 is only resistant to tigecycline and colistin, with intermediate resistance to gentamicin and tobramycin, whereas PM2 is resistant to a greater number of molecules, including ampicillin, amoxicillin/clavulanic acid, cefuroxime, and fosfomycin, which are some of the most common molecules that are used in the pharmacological treatment of UTIs. On the other hand, it has previously been shown that in bladder epithelial cells that PM1 causes acute infection with a strong inflammatory response, whereas PM2 causes a chronic infection (Fusco et al., 2017). It is also known that antibiotic resistance is one of the main aspects related to chronicisation (Beceiro et al., 2013; Perletti et al., 2013).

Uropathogenic strains can produce various virulence factors that serve in the pathogenesis of disease by facilitating the invasiveness of bacteria or the dissemination of toxins through tissues (Beceiro et al., 2013).

Between these, metalloprotease ZapA of *P. mirabilis* was considered as a virulence factor due to its ability to degrade IgA and antimicrobial peptides in a specific way (Belas et al., 2004) as well as the fact that it is mainly expressed by the swarmer phenotype (Schaffer and Pearson, 2015), which is associated with the virulence of *P. mirabilis* in the urogenital tract (Fusco et al., 2017).

The results obtained by the RT-PCR amplification analysis show that the expression of the *ZapA* gene is greater than 80% in PM1 over PM2, and this finding is confirmed by the fact that, as previously demonstrated, PM1 is associated with the swarmer phenotype (Fusco et al., 2017). Furthermore, also the protease activity on skim milk agar is much more evident in PM1 than in PM2, confirming a higher expression of ZapA. In addition, molecular fingerprinting by RAPD-PCR revealed that the two strains, even though they belong to the same species, exhibit a significantly different banding pattern, thereby confirming their different biochemical characteristics.

In the second part of our work, we analysed the ability of PM1 and PM2 to induce apoptosis in prostate cancer cells.

Our results indicate that after 18 h of incubation, PM2 induces cell death in 70% of the cells, whereas PM1 is not capable of inducing cell death, which provides further confirmation on the data related to the antibiotic resistance.

Apoptosis is a genetically regulated cell death that is and caused by multi-signal pathways that involve an energy-dependent cascade of molecular events. It is known that there are two main apoptotic pathways: the extrinsic (or death receptor) pathway and the intrinsic (or mitochondrial) pathway. The extrinsic signalling pathways involve death receptors (DRs) that are members of the tumour necrosis factor (TNF) receptor gene superfamily (Wiensa and Glenney, 2011). The best-characterised ligands and their corresponding death receptors include FasL/FasR, TNF-α/TNFR1, Apo3L/DR3, Apo2L/DR4, and Apo2L/DR5 (Ikner and Ashkenazi, 2011; Kaufmann et al., 2012; Dai et al., 2015). The binding of a DR ligand to a DR causes monomeric procaspase-8 dimerisation and activation along with the start of the apoptotic process.

The intrinsic signalling pathway involve different non-receptor-mediated stimuli that produce specific intracellular signals causing changes in the inner mitochondrial membrane, resulting in the formation of the mitochondrial permeability transition (MPT) pore, loss of the mitochondrial transmembrane potential, and the release of two main groups of proteins along with the activation of the caspase-9-dependent mitochondrial pathway. The regulation of these apoptotic mitochondrial events is controlled by the members of the Bcl-2 proteins family that are regulated by the tumour suppressor protein p53 and can be either pro-apoptotic or anti-apoptotic (Hardwick and Soane, 2013). Some of the anti-apoptotic proteins include Bcl-2, Bcl-x, Bcl-XL, Bcl-XS, Bcl-w, and BAG, whereas some of the pro-apoptotic proteins include Bcl-10, Bax, Bak, Bid, Bad, Bim, Bik, and Blk.

Both extrinsic and intrinsic pathways converge in the final phase of the apoptotic process, named execution pathway. This phase involves the activation of the execution caspases, that in turn activate cytoplasmic endonuclease and proteases, which degrade, respectively, the nuclear material and cytoskeletal proteins. Caspase-3, caspase-6, and caspase-7 work as effector or “executioner” caspases, cleaving various substrates that ultimately bring about the morphological and biochemical changes that can be seen in apoptotic cells (McIlwain et al., 2013), such as the disruption of the cytoskeleton, intracellular transport, cell division, and signal transduction (Elmore, 2007; McIlwain et al., 2013).

The analysis of the expression of apoptosis factors revealed that PM1 activates the intrinsic pathway, in general, and induces strong upregulation of Bcl-2, an anti-apoptotic factor, in particular, whereas the proapoptotic genes BAX, p53, and caspase-9 are moderately modulated. PM2, on the other hand, induces a significant increase in the expression levels of the proapoptotic genes of the extrinsic pathway, such as TNF-α/TNF-R, Fas-L/Fas-R, and caspase-8, whereas caspases-3 and -6 that are involved in the execution pathway are upregulated by both strains, even though PM2 mostly does it. This profile would explain the different response of PC-3 cells, in which, after 18 h of infection, the percentages of cellular mortality of 10% with PM1 and 75% with PM2 are observed.

Detailed knowledge of the correlations between antibiotic-resistant spectrums, the production of virulence factors, and the molecular mechanisms underlying the pathogenesis of infections are of crucial importance in making a correct diagnosis and setting up a direct and effective therapy for recurrent UTIs.

## Author Contributions

AF and GD designed the study. AF, VS, ADF, and AT were in charge of laboratory procedures. AF and ADF wrote the manuscript. All authors read and approved the final manuscript.

## Conflict of Interest Statement

The authors declare that the research was conducted in the absence of any commercial or financial relationships that could be construed as a potential conflict of interest.
